# Daily Variation in Global and Local DNA Methylation in Mouse Livers

**DOI:** 10.1371/journal.pone.0118101

**Published:** 2015-02-17

**Authors:** Lin Xia, Shihong Ma, Ying Zhang, Tao Wang, Mengyi Zhou, Zhongqiu Wang, Jianfa Zhang

**Affiliations:** 1 Center for Molecular Metabolism, Nanjing University of Science & Technology, Nanjing, 210094, China; 2 Nanjing Institute for the Comprehensive Utilization of Wild Plant, Nanjing, 210042, China; 3 Department of Biochemistry and Molecular Biology, Jiangsu University School of Medicine, Zhenjiang, 212013, China; 4 Department of Radiology, Nanjing University of Chinese Medicine, Nanjing, 210000, China; University of Lübeck, GERMANY

## Abstract

DNA methylation is one of the best-characterized epigenetic modifications and has an important biological relevance. Here we showed that global DNA methylation level in mouse livers displayed a daily variation where the peak phases occurred during the end of the day and the lowest level at the beginning of the day in the light-dark or dark-dark cycles. Typical repeat sequence long interspersed nucleotide element-1 (LINE-1) had a similar methylation rhythm to global DNA. DNA methyltransferase 3A (DNMT3A) and ratio of S-adenosylmethionine (SAM) to S-adenosylhomocysteine (SAH) brought a relative forward daily variation to global DNA methylation, and the temporary change in ratio of SAM to SAH had no influence on the DNA methylation level. The rhythm of global DNA methylation was lost and DNA methylation level was increased in *Per1^-/-^Per2^-/-^* double knockout mice, which were in accordance with changes of *Dnmt3a* mRNA levels and its rhythm. Our results suggest that the daily variation in global DNA methylation was associated with the change of *Dnmt3a* expression rather than ratio of SAM to SAH.

## Introduction

Genomic information is encoded not only by DNA sequence but also by epigenetic modifications. DNA methylation is one of the epigenetic modifications in the mammalian [1] and is mediated by DNA methyltransferases (DNMTs), which transfer a methyl group from S-adenosylmethionine (SAM) to the C5 position of cytosine in DNA [2]. Cytosine methylation is implicated in various biological and developmental processes such as genomic imprinting, X-chromosome inactivation and tissue-specific regulation of many genes, and has been shown to be essential for normal mammalian development [3]. Aberrant DNA methylation patterns, including global hypomethylation, gene-specific hypermethylation or hypomethylation, and loss of imprinting, are common in cancer tissues. Previous studies have reported that global DNA hypermethylation is associated with inflammation and increased mortality in chronic kidney disease [4]. Genome-wide changes in DNA methylation may, in particular, affect those repetitive DNA sequences that are comparatively rich in CpG dinucleotides such as long interspersed nucleotide element-1(LINE-1), short interspersed nucleotide element (SINE) represented by Alu elements in human and B1 elements in mouse and certain satellite sequences [5,6]. The methylation levels of both LINE and SINE have been reported to be a good indicator of cellular 5-methylcytosine (5-mC) level [7,8].

In mammals, DNA methylation is catalyzed mainly by three DNA methyltransferases, namely DNMT1, DNMT3A and DNMT3B [9,10]. DNMT1 has a high preference for hemimethylated DNA and copies pre-existing methylation patterns onto the new DNA strand during DNA replication [1,11]. DNMT3A and DNMT3B modify both unmethylated and hemimethylated DNA and are mainly responsible for *de novo* methylation at previously unmethylated CpG sites [1,11]. Abnormal hypermethylation in the tumorigenesis and development of prostate cancer, with higher expression of DNMT proteins compared to normal prostate tissue, has been established [12,13,14]. Previous studies have demonstrated a robust decrease in the global content of DNA methylation and a reduction of *Dnmt1* and *Dnmt3b* mRNA levels in Systemic lupus erythematosus [15,16]. These findings suggest *Dnmts* expression play an important role in regulation of DNA methylation. DNMTs belong to SAM-dependent methyltransferases, and in the reaction, SAM is converted to S-adenosylhomocysteine (SAH). SAH is hydrolyzed by S-adenosylhomocysteine hydrolase (AHCY) to homocysteine and adenosine in a reversible reaction. S-adenosylhomocysteine hydrolase-like protein 1 (AHCYL1) is member of AHCY family, and has a domain homologous to AHCY [17]. Unlike AHCY, AHCYL1 does not have hydrolase activity for SAH due to loss of key conserved residues in the critical enzymatically active site [18]. SAH is a potent inhibitor of most SAM-dependent methyltransferases including DNMTs [19], and the ratio of SAM/SAH is frequently used as an indicator of cellular methylation capacity, whereby a decrease in this ratio predicts reduced cellular methylation activity. However, two reported cases of human AHCY deficiency indicated that SAH was elevated in plasma, but leukocyte DNA was hypermethylated [20,21]. So, in the face of high SAH, even altered SAM/SAH ratio, global DNA may be methylated to a normal extent or even hypermethylated. Efforts in mammalian systems have been fueled by the notion that if there are DNMTs that methylate DNA, then there must be DNA (5-mC) demethylases that remove the methyl groups. Indeed, the existence and the nature of mammalian DNA demethylases has been the recurrent subject of uncertainty and controversy. Some demethylases, such as MBD2 and GADD45a, are controversial [22]. Recently, ten-eleven translocation (TET) family proteins have been shown to convert the covalent epigenetic mark 5-mC to 5-hydroxymethylcytosine (5-hmC) in DNA [23]. This newly discovered conversion of 5-mC to 5-hmC by TET family proteins is so far the most important and consistent mechanism underlying the active demethylation of DNA. TET proteins can further oxidize 5-hmC to 5-formylcytosine and 5-carboxylcytosine, which could eventually be removed from the genome [24].

Circadian rhythms are an evolutionarily conserved property of many biological processes in diverse life forms [25]. Many physiological and behavioral functions follow a circadian rhythm. In mammals, the circadian system is composed of both central and peripheral oscillator [26]. The central clock in the mammalian suprachiasmatic nuclei (SCN) regulates rhythms in physiology and behavior [27]. Peripheral clocks are present in almost all other tissues, such as liver, heart, and kidney where they maintain circadian rhythms and regulate tissue-specific gene expression. Mutation of clock genes leads to abnormal circadian rhythms of locomotor activity. It is observed that double *Per1/Per2* mutants lead to complete arrhythmicity [28,29]. Previous studies have reported that DNA methylation in human blood shows 24-h variation [30], and altered *Bmal1* expression affects the DNA methylation state [31]. In this study, we found that global DNA methylation level in mouse livers displayed a daily variation pattern, which was associated with the expression of *Dnmts* rather than the SAM/SAH ratio.

## Materials and Methods

### Animals

8-week-old male C57BL/6 wild-type (WT) and *Per1* ^*-/-*^ *Per2* ^*-/-*^ double knockout (DKO) mice were used in this work. Animals were maintained in 12/12 light/dark (LD) cycles with light on at 7:00 am and off at 7:00 pm, and given food and water *ad libitum*. Some WT mice were transferred into dark/dark (DD) cycles for 48 hours. All procedures were approved by the institutional Animal Care and Use Committee at Nanjing University of Science and Technology. All surgery was performed under sodium pentobarbital anesthesia, and all efforts were made to minimize suffering.

### Analysis of 5-Methylcytosine DNA Content

Genomic DNA was isolated from liver tissue by standard phenol-chloroform extraction, and digested to individual bases by incubation in hydrofluoric acid at 80°C for 4h as previously described [32]. The 5-mC content was determined according to the method described previously with some modifications [33]. Briefly, the hydrolysates were dissolved in 0.1 mL of HPLC mobile phase and applied to a Partisphere bonded phase C18 (reverse phase) cartridge column eluted with 20 mM ammonium phosphate (pH 2.3), at a flow rate of 1mL/min, using Waters 1525 System (Millipore Corp., Bedford, MA). The DNA bases were identified by comparing with retention time of standards at 280 nm. Cytosine and 5-mC standards were purchased from Sigma (St. Louis, MO).

### Bisulphite conversion and LINE-1 methylation analysis

Genomic DNA was isolated from liver tissue by standard phenol-chloroform extraction, and sodium bisulfite conversion was carried out as previously described [34]. Quantitative LINE-1 methylation analysis was performed by bisulfite sequencing PCR (BSP) [35]. Primers for the LINE-1 element were designed to amplify nucleotides 64–326 of the consensus sequence which contain 15 CpG dinucleotides [36]. Amplified DNA was ligated into pCR2.1 vector (Invitrogen, Carlsbad, CA) and transformed into competent *E. coli* (DH5α). 10 clones were selected and sequenced with M13F primer or M13R primer. The results were analyzed by Biq Analyzer software.

### Treatment with 5′-AMP

The indicated doses of 5′-AMP were solvated in 0.01 M phosphate-buffered saline (PBS), pH 7.2 and administered to mice by intraperitoneal (i.p.) injection at 9:00 am. The same volume of PBS was injected as a control. One hour and four hours after injection, mice were sacrificed by cervical dislocation and livers were removed and freeze-clamped in liquid nitrogen.

### Determination of hepatic levels of adenosine, SAM and SAH

Adenosine, SAM and SAH were extracted from frozen samples using 0.4 N perchloric acid and analyzed by reverse-phase HPLC (Waters 1525 system; Millipore Corp., Bedford, MA), according to the procedure previously described with some modifications [37]. The mobile phase contained 0.1 M sodium acetate, 5 mM heptanesulfonic acid adjusted to pH 4.5 with acetic acid, and 5.5% acetonitrile. The samples were eluted at room temperature with an invariable gradient at a flow rate of 0.8 mL/min. Characteristic peak spectra and retention times compared with those of the standards were used to identify adenosine, SAM and SAH. Quantitation was based on peak areas. Adenosine, SAM and SAH standards were purchased from Sigma (St. Louis, MO).

### Quantitative real-time RT-PCR and Western blotting analysis

Total RNA from liver sample was extracted using Trizol (Invitrogen, Carlsbad, CA) according to the manufacturer’s instruction. Reverse transcript reaction was carried out by Invitrogen reverse transcript enzyme following the manufacturer’s protocol. Quantitative real-time RT-PCR was performed, and the results were analyzed using an ABI 7300 Detection System in combination with SYBR green dye. The primer sequences are shown in Table 1. Relative gene expression compared with *Gapdh* expression was calculated by the comparative threshold cycle method. Western blotting assay were performed as previously described [38]. Anti-DNMT3A (#3598) antibodies were obtained from Cell Signalling Technology. Quantification of the bands was performed using Gel Analysis V2.02 software (Clin Science Instruments, China).

**Table 1:** Primer sequences for real-time RT-PCR analysis.

Gene	Primer Sequences (5′- 3′)
Gapdh	Forward: CATCCACTGGTGCTGCCAAGGCTGT
	Reverse: ACAACCTGGTCCTCAGTGTAGCCCA
Dnmt1	Forward: CTACCTGGCTAAAGTCAAGTC
	Reverse: CACTCTCTGTGTCTACAACTC
Dnmt3a	Forward: GCACCTATGGGCTGCTGCGAAGACG
	Reverse: CTGCCTCCAATCACCAGGTCGAATG
Dnmt3b	Forward: CAAGGAGGGCGACAACCGTCCATT
	Reverse: TGTTGGACACGTCCGTGTAGTGAG
Tet2	Forward: AACCTGGCTACTGTCATTGCTCCA
	Reverse: ATGTTCTGCTGGTCTCTGTGGGAA
Tet3	Forward: TCCGGATTGAGAAGGTCATC
	Reverse: CCAGGCCAGGATCAAGATAA

### Statistical analyses

Data are presented as mean ± SEM. Statistical analysis was performed with one-way or two-way ANOVA with LSD post hoc test and a Student’s *t-test*. One-way ANOVA was used to verify significant effects of circadian time within each genotype. Two-way ANOVA was used to identify differences between the genotypes. The circadian variation was also assessed by the single cosinor method [39]. The rhythm characteristics estimated by this method included the mesor (middle value of the fitted cosine representing a rhythm-adjusted mean), the amplitude (half the difference between the minimum and maximum of the fitted cosine function), and the acrophase (time of peak value in the fitted cosine function). Significance was defined as *p* < 0.05.

## Results

### 5-methylcytosine content showed a significant rhythm in mouse liver

**Fig 1:**
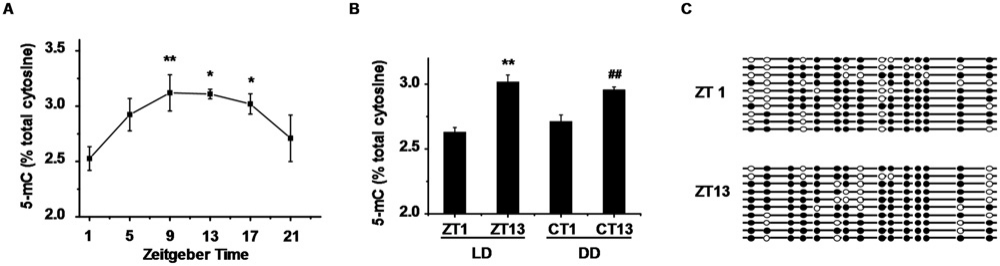
Daily variation of global DNA 5-methylcytosine content in WT mice. (A) One-way ANOVA showed significant variation of genomic 5-mC content over time in livers of WT mice (*p* = 0.048). Data represent means ± S.E.M. (n = 4). *p < 0.05, **p < 0.01 for LSD post hoc test compared with ZT1. (B) Genomic 5-mC content was analyzed at CT1 and CT13 in DD cycles. Data represent means ± S.E.M. (n = 4). **p < 0.01 compared with ZT1, ^##^ *p* < 0.01 compared with CT1. (C) Increased 5-methylcytosine content in LINE-1 at ZT13 vs. ZT1 in WT mice. 64–326 of the consensus sequence in LINE-1 containing 15 CpG dinucleotides were analyzed. Each line represents a unique DNA clone; filled and open circles represent methylated and unmethylated CpGs, respectively. ZT: zeitgeber time, CT: circadian time.

The genomic 5-mC content from WT mice liver was examined at zeitgeber times (ZTs) of 1, 5, 9, 13, 17 and 21 (ZT0 corresponds to light on and ZT12 to light off) by HPLC, and significant 24-h variations in global DNA methylation level were observed (Fig. 1A). The peak phases occurred during the end of the day and the lowest level at the beginning of the day, which was also present in mice in dark-dark cycles (Fig. 1B). Interspersed repetitive sequences have mainly been used to quantify genome-wide methylation measurements. Among these sequences, LINE-1 sequences have been frequently studied. We then investigated the level of LINE-1 methylation at ZT1 and ZT13. LINE-1 methylation was lower at ZT1 compared with at ZT13 (Fig. 1C), and the average percentage of methylated cytosines in LINE-1 sequences were 72.0% ± 2.9% at ZT1 and 79.3% ± 2.9% at ZT13 (*p* = 0.046).

### Patterns of SAM/SAH ratio and SAH concentration in mouse livers

**Fig 2:**
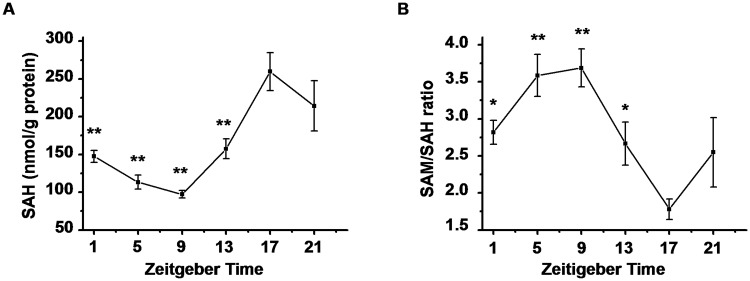
Daily changes of SAH concentration and SAM/SAH ratio in WT liver. One-way ANOVA showed that both (A) SAH and (B) SAM/SAH ratio displayed a clear diurnal variation (*p* < 0.01). Data represent means ± S.E.M. (n = 4). *p < 0.05, **p < 0.01 for LSD post hoc test compared with ZT17.

**Fig 3:**
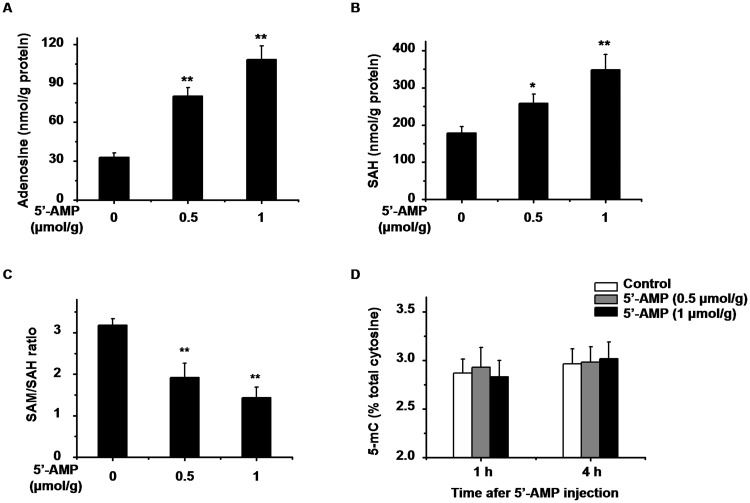
Effect of administration of 5′-AMP on global DNA methylation. HPLC analysis for the level of (A) adenosine, (B) SAH, (C) SAM/SAH ratio at 1 h after i.p. injection of 5′-AMP (0.5μmol/g and 1μmol/g body weight). (D) Genomic 5-mC content at 1 h and 4 h after 5′-AMP treatment. Data represent means ± S.E.M. (n = 4). *p < 0.05, **p < 0.01 compared with saline control.

The ratio of SAM to SAH, also called methylation potential, is a metabolic indicator for cellular methylation status. SAH, a methyltransferases inhibitor, is itself considered a predictor of reduce methylation capacity. HPLC analysis showed that intracellular SAH concentration and SAM/SAH ratio displayed a clear diurnal variation (Fig. 2A and 2B). The acrophase of the ratio occurred about 4 hours earlier than methylation, and the pattern of SAH was inversely correlated with the ratio. Previous investigation demonstrated that adenosine 5′-monophosphate (5′-AMP) injection increased intracellular adenosine level [40]. Similarly, at 1 h after intraperitoneal injection, 5′-AMP caused a dose-dependent increase of adenosine level in the liver (Fig. 3A); the level of SAH was increased and the ratio of SAM to SAH was decreased markedly (Fig. 3B and 3C). The global DNA methylation levels were not changed at 1 h or 4 h after 5′-AMP injection (Fig. 3D). These results suggested that the daily variation in global DNA methylation was not directly influenced by SAM/SAH ratio.

### *Dnmts* and *Tets* expression pattern in mouse livers

**Fig 4:**
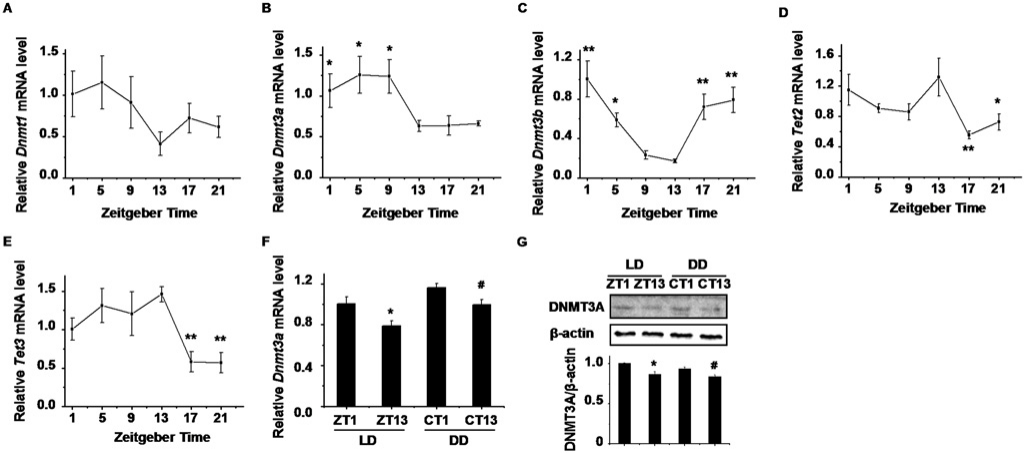
Hepatic expression of *Dnmts* and *Tets* in WT mice. The mRNA expression of (A) *Dnmt1*, (B) *Dnmt3a*, (C) *Dnmt3b*, (D) *Tet2* and (E) *Tet3* in WT livers. One-way ANOVA showed significant variations of the expression of *Dnmt3a*, *Dnmt3b*, *Tet2* and *Tet3* over time in liver (*p* < 0.05) and no significant variations in *Dnmt1* (*p* > 0.05). Data represent means ± S.E.M. (n = 3–4). *p < 0.05, **p < 0.01 for LSD post hoc test compared with ZT13. (F) The hepatic *Dnmt3a* mRNA levels in mice under LD and DD. Data represent means ± S.E.M. (n = 4). (G) Western blotting analysis for DNMT3A protein levels. The signal intensities of DNMT3A were normalized to the intensities of β-actin. Data represent means ± S.E.M. from three independent experiments. *p < 0.05 compared with ZT1, ^#^ *p* < 0.05 compared with CT1. ZT: zeitgeber time, CT: circadian time.

DNA methylation is regulated by DNMTs which are involved in *de novo* and maintenance methylation, so we examined *Dnmts* mRNA level. While the *Dnmt1* mRNA level had no 24-h variation (Fig. 4A), both *Dnmt3a* and *Dnmt3b* mRNA levels displayed a significant daily variation in the livers of WT mice (Fig. 4B and 4C). A broad peak of *Dnmt3a* mRNA levels was confirmed from ZT1 to ZT9, with peak/trough ratio of 2. The mRNA of *Dnmt3a* tended to accumulate more in the light phase, and its rhythm was in accordance with the variations in global DNA methylation level. The highest expression of *Dnmt3b* appeared at ZT1 and the lowest around ZT13 with 5-fold peak/trough ratio, which inversely correlated with the level of DNA methylation. Next, we examined DNA demethylase *Tets* mRNA levels. Both *Tet2* and *Tet3* mRNA levels displayed a significant daily variation in WT mice liver (Fig. 4D and 4E). The expression of *Tet2* was decreased at ZT17 to form a trough and *Tet3* mRNA levels were also reduced at ZT17 and ZT21, which was not related to daily changes of the level of DNA methylation. Then, we investigated *Dnmt3a* mRNA and protein levels in livers of mice in dark-dark cycles. The results revealed that both *Dnmt3a* mRNA and protein levels were decreased at subject night time compared with that at subject light time, which was accordant with the observation in light-dark cycles (Fig. 4F and 4G)

### Effect of *Per1* ^*-/-*^ *Per2* ^*-/-*^ double knockout on the patterns of daily variations

**Fig 5:**
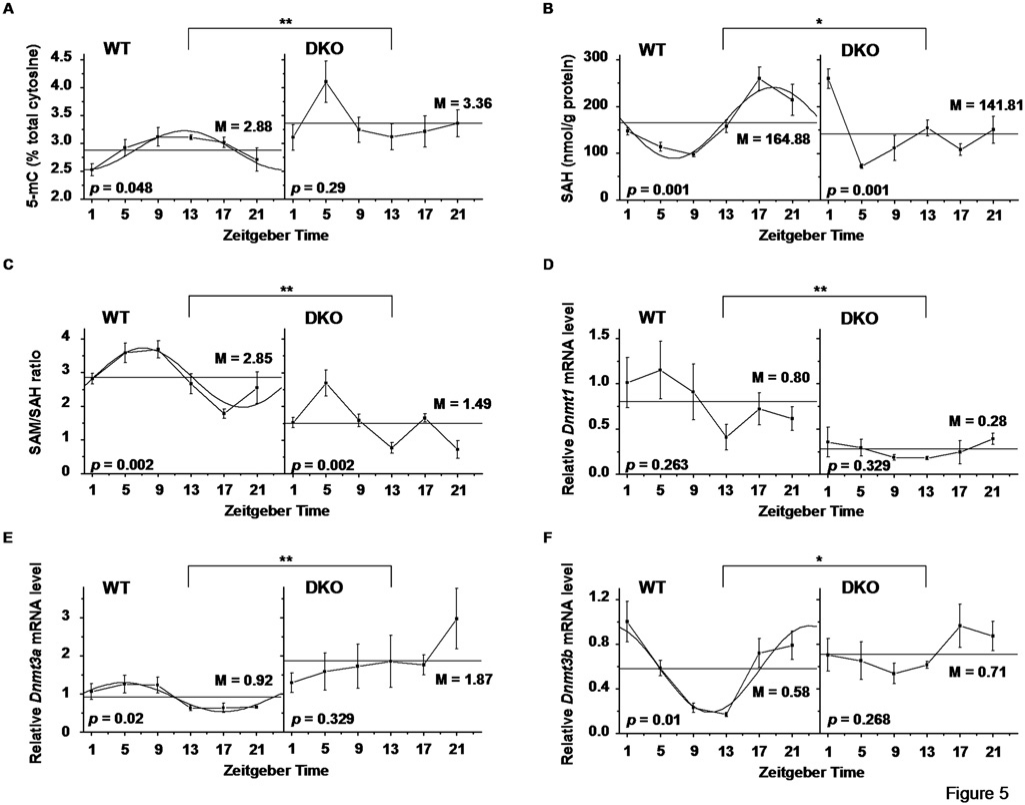
Effect of *Per1* ^*-/-*^ *Per2* ^*-/-*^ DKO on the patterns of daily variations. (A) Genomic 5-mC content, (B) SAH, (C) SAM/SAH ratio, and the expression of (D) *Dnmt1*, (E) *Dnmt3a* and (F) *Dnmt3b* were analyzed by single cosinor method in WT and DKO mice. Data represent means ± S.E.M. (n = 3–4). *P* value from time effect by one-way ANOVA analysis of variance. *p < 0.05, **p < 0.01 by two-way ANOVA comparing WT and DKO mice. M: mesor.

Knockout mice lacking either *Per1* or *Per2* have an altered and sometimes unstable free-running circadian period. In *Per1* ^*-/-*^ *Per2* ^*-/-*^ DKO mice, circadian rhythmicity can not be maintained [28,29]. We then analyzed that the daily variations of global DNA methylation (Fig. 5A), SAH level (Fig. 5B), SAM/SAH ratio (Fig. 5C), and *Dnmts* mRNA expression (Fig. 5D, 5E and 5F) in the livers of WT and *Per1* ^*-/-*^ *Per2* ^*-/-*^ DKO mice. Two-way ANOVA analysis showed there were significant different variations in these factors between WT and DKO mice. As expectedly, lacking *Per1* and *Per2* lost circadian rhythms in 5-mC content, SAH, SAM/SAH ratio and *Dnmts* mRNA expression. The analysis with the single cosinor method revealed that the mesor of 5-mC content in DKO livers was significantly higher than that in WT livers (Table 2). Interestingly, in DKO livers, while the circadian rhythms of *Dnmt3a* were lost, the mesor of *Dnmt3a* mRNA expression was higher compared with WT livers (Table 2). These results reflected a special relationship between *Dnmt3a* mRNA and daily variation of 5-mC.

**Table 2:** Circadian rhythmic parameters of 5-methylcytosine content, SAH concentration, SAM/SAH ratio and DNMT gene transcriptions in livers of WT and *Per1^-/-^Per2^-/-^* DKO mice.

	Mesor		Amplitude		Acrophase ZT (h)	
	WT	DKO	WT	DKO	WT	DKO
5-mC	2.88 ± 0.12	3.36 ± 0.23*	0.35 ± 0.04	—	12.13 ± 0.69	—
SAH	164.88 ± 14.95	141.81 ± 19.49	76.09 ± 12.32	—	18.72 ± 1.45	—
SAM/SAH ratio	2.85 ± 0.26	1.49 ± 0.20**	0.88 ± 0.08	—	7.09 ± 1.09	—
Dnmt1	0.80 ± 0.20	0.28 ± 0.09*	—	—	—	—
Dnmt3a	0.92 ± 0.14	1.87 ± 0.52*	0.38 ± 0.09	—	4.91 ± 1.09	—
Dnmt3b	0.58 ± 0.09	0.71 ± 0.12	0.39 ± 0.07	—	23.73 ± 0.03	—

Data represent means ± S.E.M. (n = 3–4).

*p < 0.05,

**p < 0.01 compared with WT.

## Discussion

It is well known that DNA methylation plays a critical role in gene regulation and has been implicated in the etiology of chronic disease including atherosclerosis, neural degeneration and cancer [41,42]. In the present study, we observed that global hepatic DNA methylation level displayed a daily circadian variation in LD and DD cycles, which was impaired in *Per1* ^*-/-*^ *Per2* ^*-/-*^ DKO mice. Even if under normal light-dark cycles, DKO mice are completely arrhythmic [28,29], displaying a disorder change in multiple genes expression and physiological patters. 5-mC content in LINE-1, which may be affected by genome-wide changes in DNA methylation, displayed a similar change to global DNA methylation. By analyzing hepatic promoter DNA methylation levels between peak and trough times of transcription, circadian oscillation in liver DNA methylation is consider as either very rare or small in amplitude [43], and strand-specific methylation/demethylation occurs during transcriptional cycling of promoter [44]. Thus, the variation of methylation of repetitive DNA sequences could represent daily variation of global DNA methylation.

DNA methylation may be affected by a limited availability of SAM or an increase in SAH, the SAM/SAH ratio is often a predictor for methylation. As previous observation [45], both SAM/SAH ratio and SAH concentration displayed an obvious daily rhythm, implying an alteration of SAM/SAH ratio could correlate with the daily variations of global DNA methylation. However, we did not find any changes of global DNA methylation level in different doses of 5′-AMP injection, while the accumulation of intracellular adenosine led to an increase of SAH and a reduction of SAM/SAH ratio. It is reported that the reduced methylation potentials fail to change the global DNA methylation in HepG2 cell [46]. However, intraventricular adenosine-releasing silk decreases hippocampal DNA methylation in naive rats 5 days after implantation [47]. It suggests a temporary change in ratio of SAM to SAH could not influence global DNA methylation level.

DNMT3A and DNMT3B are essential for *de novo* methylation and regulate levels of global DNA methylation [48]. We found the expression of *Dnmt3a* displayed a strong consistent with global DNA methylation, while *Dnmt3b* expression was inversely correlated with the level of DNA methylation. *Dnmt1* expression displayed no significant rhythm over 24 h in livers. It is known that changes of DNA methylation in SCNs are visible in mice exposed to different day lengths, and one plausible mechanism would be light-dependent induction or repression of the enzymes catalyzing DNA methylation and demethylation [49], and that methylation with age is decreased in mice, rats and human, and occurs in the brain, liver, heart, and spleen [50,51,52]. Moreover, *Dnmt1* and *Dnmt3a* express at significantly higher levels in young group and decrease with age [53], implying an interrelation between global DNA methylation and *Dnmts*. In our observation, DNMT3A mRNA and protein levels were increased in subject day time compared with in subject night time, which was in accordance with the daily changes of 5-mC. DNMT3A and DNMT3B possess deaminase activity and propose that both enzymes are involved in a dynamic demethylation-methylation pathway that operates during gene transcription [44], and it is reported that the quantitative difference of DNMT3B protein between ZT0 and ZT8 could not be found by western blotting analysis [31]. Mice lacking *Per1/Per2* results in complete arrhythmicity [28,29]. The rhythms of *Dnmt3a* expression and DNA methylation levels were lost in livers of *Per1/Per2* double knockout mice, in which the levels of both DNA methylation and *Dnmt3a* expression increased accordantly. It is known that DNMT3A and DNMT3B are de novo methyltransferases and show similar activity on unmethylated and hemimethylated DNA [54]. DNMT3A may methylate some CpG sites more frequently than others, depending on the sequence context [55], and the certain CpG sites within genes locus are preferentially methylated by DNMT3A but not by DNMT3B [56].

In conclusion, our observations revealed that circadian system modulates the daily variation in global and local DNA methylation, and this variation is associated with the changes of *Dnmt3a* expression rather than SAM/SAH ratio.
